# A Review of Five Existing Hornet-Tracking Methods

**DOI:** 10.3390/insects15080601

**Published:** 2024-08-10

**Authors:** Chengzhi Wang, Jiaxing Huang, Xia Wan, Zhanbao Guo

**Affiliations:** 1 State Key Laboratory of Resource Insects, Institute of Apicultural Research, Chinese Academy of Agricultural Sciences, Beijing 100093, China; x22301090@stu.ahu.edu.cn (C.W.); huangjiaxing@caas.cn (J.H.); 2 Key Laboratory for Insect-Pollinator Biology of the Ministry of Agriculture and Rural Affairs, Institute of Apicultural Research, Chinese Academy of Agricultural Sciences, Beijing 100093, China; 3 Department of Ecology, School of Resources and Engineering, Anhui University, Hefei 230601, China; 4 Anhui Province Key Laboratory of Wetland Ecosystem Protection and Restoration, Hefei 230601, China

**Keywords:** hornet, tag tracking, triangulation, thermal imaging, harmonic radar, radio telemetry

## Abstract

**Simple Summary:**

In recent years, with global warming and climate change, there have been many cases of successful hornet invasions in some areas. The successful invasion of hornets not only has an impact on local human security and economic activities, but also seriously threatens the bee ecosystem, which has attracted the attention of scientists. Therefore, taking some measures to suppress the foraging and expansion of hornets becomes an important subject. Compared with attractant trapping and poison baits, hornet-tracking technology, which tracks hornets to find their nests, is a more efficient and feasible method. This review paper discusses several common hornet-tracking methods, analyzes the advantages and disadvantages of different methods, and provides a theoretical basis for the development of hornet-tracking technology.

**Abstract:**

Hornet is a general term for insects of the genus *Vespa* (Hymenoptera: Vespidae). Hornets are predatory insects distributed worldwide. They often appear at apiaries in groups to prey on honey bees, and cause incalculable losses in the honey bee industry. In the face of hornet intrusion, tracking a homing hornet to find its nest is the most efficient way to discover and eliminate the hornets around an apiary. Here, five hornet-tracking methods (hornet tag tracking, triangulation, thermal imaging technology, harmonic radar, and radio telemetry) are reviewed. The advantages, disadvantages and feasibility of each method are discussed to improve the strategies for tracking hornets. Therefore, this review provides ideas for the development of hornet-tracking technology and for improving honey bee protection.

## 1. Introduction

Hornets belong to the genus *Vespa* (Hymenoptera: Vespidae) and are predatory eusocial insects that form moderate to large annual colonies [1], with 22 extant hornet species known worldwide [1,2]. Some invasive species of hornets have caused significant pressure on human health and social activities in some areas [3]. At the same time, their predation of pollinating insects negatively impacts on local pollination services [4]. For example, they prey on bumblebees (*Bombus terrestris* Linnaeus, 1758) and honey bees [5], and their predation of the latter has severely affected the honey bee industry in many parts of the world [6,7]. Among them, yellow-legged hornets (*Vespa velutina* Lepeletier, 1836), Asian giant hornets (*Vespa mandarinia* Smith, 1852), and Oriental hornets (*Vespa orientalis* Linnaeus, 1771) have captured the most attention [1]. The yellow-legged hornet is native to Southeast Asia [8,9]. However, it is an invasive species in East Asia and Central and Western Europe [10,11]; it has rapidly spread throughout Europe since its first discovery in France in 2004, causing severe damage to local honey bee colonies [12,13]. Similarly, the Asian giant hornet was detected in western British Columbia in Canada and Washington State in the United States [6,14], and it may have successfully wintered in North America [15]. Over the past few years, *V. orientalis* has colonized several European countries [16]. In recent years, the Oriental hornet was found in central Chile in 2020 [17], posing a direct threat to the beekeeping industry and having the potential for continued invasion.

The invasion of apiaries by hornets has always been a problem for beekeepers, and the economic losses caused are difficult to estimate. Generally, newly emerged hornet queens mainly feed on sugar sources such as nectar and fruit juice, but brooding requires them to consume a large amount of protein to ensure the growth of larvae [18,19]. To obtain protein, hornet workers hunt a wide range of insects, and honey bees are among their most preferred protein sources [19,20]. The identification of prey pellets revealed that the proportion of honey bees among the prey captured by yellow-legged hornets reached 38.1%, which was the highest proportion of all prey [21]. They prey on bees at the entrance of the bee hive, which leads to an increase in the probability of overall homing failure of foraging bees [22,23]. This lockdown causes stress and seriously affects bee colony strength [18]. *V. mandarinia* is the largest hornet in the world [24], and this species marks the target beehive with pheromones as a food source signal to their cohort [25]. Then, they appear in groups in front of the hive to kill the worker bees and enter the hive to plunder the honey, pollen, and larvae. In Japan, there are reports that more than a dozen hornets can destroy a colony of 30,000 individuals [15]. The eastern honey bee (*Apis cerana* Fabricius, 1793) has evolved several strategies during its long struggle with hornets [5,26]. For example, *A. cerana* uses the “heat-balling” [27] defense strategy to produce high concentrations of CO_2_ and high temperature and humidity to wrap and kill its natural enemy, *V. mandarinia* [28,29]. Another strategy of *A. cerana* is to collect plant materials to smear around the hive entrance to protect against hornets [30]. However, most western honey bees (*Apis mellifera* Linnaeus, 1758) have difficulty resisting hornets because of their inefficient and disorganized defenses against them [31]. Therefore, hornet invasion has created great challenges for honey bee management.

At present, hornet control methods include attractant trapping, poisoned baits, and nest-tracking methods. The attractant trapping method requires the combination of an attractant and a trap to capture hornets and prevent the hornets from intruding the apiary [32]. Common attractants include sugar-based baits [32,33], protein baits [34], and various compounds (pheromones and chemical volatiles) [20,35]. This method can safely and effectively suppress the spread of hornets in the apiary. However, the traps attract not only hornets but also many nontarget insects [36]. The method by which hornets carry poisoned baits to destroy their nests is effective, and these poisoned baits can destroy nests remotely. However, releasing toxic substances into the environment can cause collateral damage [37]. Nest tracking utilizes the homing instinct of hornets [38,39]. Tracking the homing hornets and locating their nests may be a viable approach to contain them [40]. Of course, through social propaganda and collecting local residents’ eyewitness reports on hornets and hornet nests, combined with hornet-tracking technology, the nests can be locked more quickly and efficiently. For example, in the case of controlling *V. velutina* on a Mediterranean island, more than half of the nests (58%) were found through citizen reports [41].

The early identification and destruction of nests by tracking homing hornets is a more effective method for overcoming the current hornet crisis [39,42]. At present, the commonly used tracking technologies are visual tracking and radio signal tracking. In view of the advantages of using hornet-tracking technology for addressing hornet invasion, we summarize various hornet-tracking methods to provide a theoretical basis for the prevention and control of hornet invasion.

## 2. Visual Tracking

### 2.1. Hornet Tag Tracking

Once the hornets fly away from the apiary, they are very difficult to track with the naked eye. The hornets quickly get out of sight or blend into the complex sky background, resulting in tracking failure. A hornet tag can assist the naked eye in observing the flight of a hornet, thereby simplifying tracking. A tag is generally composed of a thin thread and a lightweight feather or paper strip. The thread is tied to the waist of the hornet as a tracking mark. The tracking mark of a hornet can be tracked with a telescope or an unmanned aerial vehicle (UAV) to locate the hornet’s nest. Reynaud and Guérin-Lassous designed a system that can achieve multiple UAVs with autonomous flight capabilities to cooperate in the tracking of tagged hornets [43].

Hornet tags are widely used in folk because of their low cost and ease of operation, but they also present many problems. In practice, we found that the size and shape of the hornet tag will affect visual tracking. Moreover, the weight and air resistance of hornet tags affect hornets’ flight. Even if a hornet can fly normally, another problem is that the hornet may become entangled with branches and leaves when it lands on vegetation, preventing it from moving forward. Of course, there is also a risk of losing the tracking target when the hornet’s flight trajectory is obscured. Turchi and Derijard proposed another issue: when hornets find that a thread is tied to its abdomen, it will stay on a nearby tree to try to remove the tag [37]. Therefore, hornet tags are suitable for use on relatively flat terrains with few trees.

Recently, a special hornet tag was proposed. Thomas Walter et al. used the photoluminescence properties of passivated lead sulfide quantum dots as luminescent tags (weight 12.5 mg; diameter 5 mm) for hornets [44]. These tags can be directly adhered to the hornet, allowing the hornet and the label to be integrated. Then, their developed shortwave infrared detection system can be used to record and determine the position of the hornet with a delay of less than 10 ms. Whether the same effect can be achieved by using light-reflecting materials or other luminescent materials remains to be further verified. Researchers have successfully tracked honey bees using a lightweight retroreflective tag. The system detects bees through a camera with a global electronic shutter, enabling 3D flight path analysis and long-term automated monitoring of honey bees within a range of 35 m [45].

### 2.2. Triangulation

**Figure 1:**
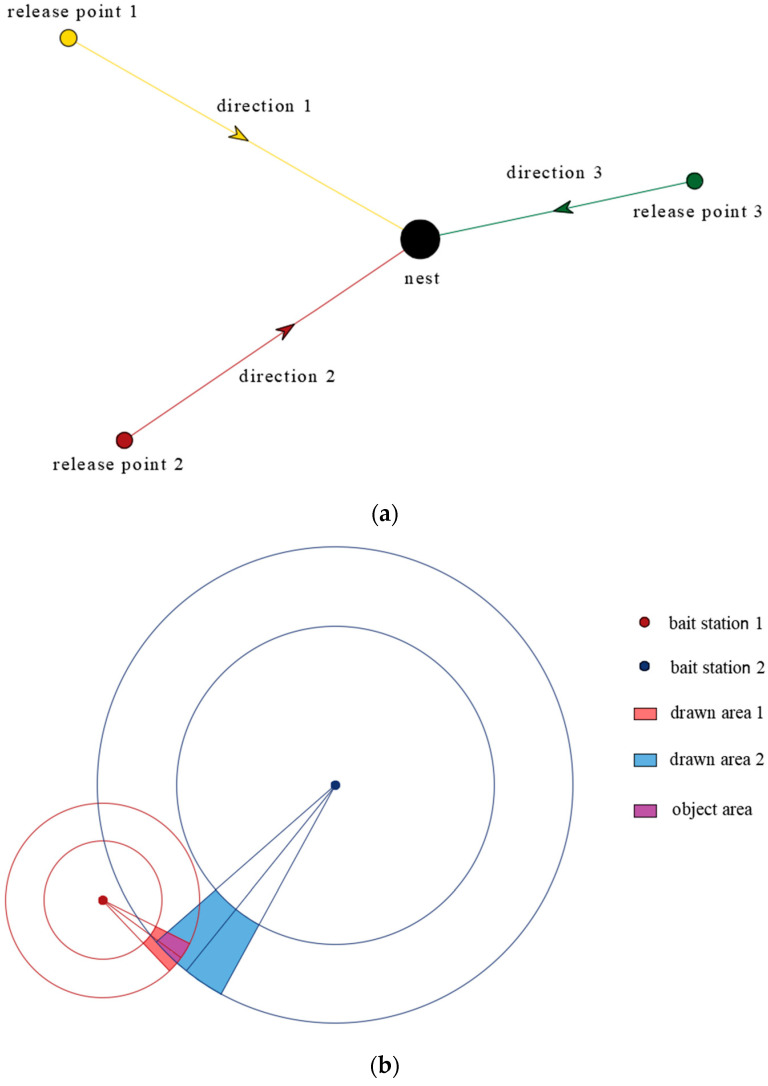
(**a**) Conventional triangulation tracking method; (**b**) The new triangulation tracking method.

Triangulation is a traditional technique used for locating hornet nests. This method involves releasing the specimens captured in at least three locations and recording the direction of their homing (Figure 1a). If the samples come from the same nest, the three directional lines converge to a point that indicates the location of the nest [37]. Leza et al. set up 87 bait stations around a hornet-invaded apiary to observe the flight routes of hornets from 2 or 3 feeding points, then drew and triangulated these flight routes on a map, and finally located the nest by visual observation [46]. Although this method can track hornet nests, it requires many experienced operators and material resources. In addition, this method will not be applicable if the released hornets belong to multiple nests.

Rojas-Nossa et al. proposed a method for detecting nests with high density distribution [47]. The method involves recording the direction in which the marked hornet leaves the bait station, and the time it takes to travel a round trip between the bait station and the nest. Then, according to the times and directions recorded, two external lines are drawn on a map, defined on both sides by the average direction line, with an angle from the average line of 10° or 15° (for times shorter than 10 min, it is 10°, for times greater than 10 min, it is 15°). Then, a scalloped area is obtained. The distance is estimated based on the time, including the shortest and longest estimated distances, and concentric circles are drawn on the map so that the concentric circles and the scalloped area intersect to form a new area that is then searched. If the nest is not found, a second bait station is built, and the same method is used to construct an area. If the area intersects with the first area, a smaller interaction area is then formed and the target area is reduced to locate the approximate position of the nest (Figure 1b).

Through this method, the target can be located within a smaller area, improving the efficiency of tracking; this approach is a simple operation that anyone can learn in a short training time, making it suitable for promotion in vast rural areas. However, some hornets stay or hover on nearby trees before homing, which is likely to cause deviations in the measurements and results. At present, there are few reports on the homing trajectory of hornets, but scientists have found that ground-nesting wasps (Sphecidae: *Cerceris australis*) conduct arc segment trajectories when homing through the view guidance in memory [48].

### 2.3. Thermal Imaging Technology

During the tracking process, once the location of the hornet nest is narrowed down to a small area, it is difficult to find the specific location of the nest with the naked eye due to occlusion by leaves or a lack of light. It has been shown that social insects, including hornets, can control the temperature of their nests [3,49,50]. Some hornets can use the insulation characteristics of their nest envelope to maintain the temperature of the nest at 28–30 °C by changing their metabolism [51,52,53]. Thermal imaging is a remote and passive monitoring technology that can detect and record infrared radiation emitted by objects [54]. The detectability of an object is proportional to the temperature difference between the object and the surrounding environment. Therefore, in theory, thermal imaging technology could be used to detect hornet nests.

Lioy et al. tested this method on three nests under different environments and operating conditions and reported that the time, distance, and presence or absence of leaf occlusion had an impact on the ability to detect thermal signals [53]. The results showed that the morning is the best time of the day for thermal imaging detection. The closer the temperature is to the average temperature of the nest, the more difficult it is to detect. The detectability improves as the distance between the operator and the nest decreases. The presence of a canopy in front of a nest also reduces the ability to detect the nest.

Hornet nests built on trees, such as those of *V. velutina*, can be detected using this technique [40,53]. However, thermal imaging has difficulty detecting nests built in hidden places. For example, the nests of Asian giant hornets are built underground [55], such as in natural caves, snake caves, or decaying tree roots, making them difficult to detect [15]. Similarly, bumblebee nest in holes [56], and using thermal imaging technology to locate wild bumblebee nests by observing nest traffic has proven to be inefficient [57]. Although insects such as hornets have certain thermostatic abilities [58], it is still difficult to detect them clearly using long-distance thermal imaging technology.

Although thermal imaging technology shows advantages in accurate positioning, there are various factors that are not conducive to detection (temperature differences, distance, and occlusion). Within a small range, thermal imaging technology can detect the surrounding environment of a bee field, and in a large area, thermal imaging can also be combined with the use of drones to search through the surrounding canopy.

## 3. Radio Signal Tracking

### 3.1. Harmonic Radar

Harmonic radar has been used to track insects since the late 1980s [59]. It is widely used in the study of farmland pests and monitoring the behavior of insects, and is a relatively mature technology [60,61,62,63]. Its working principle is to install a passive lightweight transponder (usually a Schottky diode) on the tracked insect [61]. Because harmonic radar uses the super high frequency (SHF) band, compared to the transmitters used in radio telemetry, the antenna used by the transponder is shorter (12–16 mm), and the weight of the transponder is lighter (<20 mg) [44,63,64]. The radar transmission system transmits the signal, amplifies and reflects the fundamental frequency signal after the transponder receives it, and then receives it back via the radar receiving system. The distance between the transponder and the radar can be determined by measuring the time delay between the transmitted signal and the received signal. Moreover, the direction of the object is the direction from which the receiving antenna receives the signal [42]. The location update frequency is related to the device, natural environment, and the path and speed of the tracked target [44,61], and the position update frequency is proportional to the route display accuracy. Generally, the position is updated every 3 s [65], and the resolution is (±2.5 m) [44]. Through multiple launches, the detection of the trajectory of the tracked object can be achieved. Therefore, the object can be tracked by radar.

Harmonic radar can be used to effectively track insects in low-altitude plain areas. However, it is difficult to achieve the desired effect in complex environments in mountainous areas. Harmonic radar tags rely on a reflected signal rather than a signal that is generated, so they cannot be uniquely identified. They are highly sensitive to the direction of the tag relative to the radar unit and the influence of terrain and vegetation [61]. With increasing altitude, occlusion by dense trees influences the transmission of signals [66]. As a result, the detection distance becomes shorter, or it even becomes impossible to detect the signal. The maximum detectable range of the harmonic radar is approximately 900 m in a plain unshielded area [40,67,68]. For example, the mountain forest area tested by Daniele Milanesio’s team is only 125 m long [40]. The range is not enough when compared to the average foraging range of *V. velutina* (395 ± 208 m, maximum 786 m) [69]. Moreover, some hornets can forage much farther than 1000 m [13]. The foraging distance of *V. mandarinia* is even farther (usual foraging distance = 1000 m to 2000 m; max = 8000 m) [70].

To address the effects of distance and mountainous terrain, Riccardo Maggiora et al. successfully developed a harmonic radar that can track hornets in a 500 m range over mountainous forest terrain [42]. Harmonic radar achieves a wider range of detection by changing the beam width. Its azimuth width is only 1.5° of the half-power beam width (HPBW) (the width of the HPBW is proportional to the coverage area and inversely proportional to the signal strength), while the elevation angle beam width reaches 24° HPBW, which improves the radar in the vertical direction, with a tracking range of up to 500 m. The radiating surface makes this approach more suitable for terrain with altitude variation. The team previously attempted to install the radar system on a liftable telescopic tower to improve the mobility of the radar system and the ability to quickly lock onto the flight direction of hornets [71].

The advantage of harmonic radar over other tracking methods is that it can display the position and flight path of hornets in real time. However, due to its complex system and low portability, harmonic radar is particularly cumbersome in the actual tracking process. The whole system can be installed on a car to achieve mobility. However, the complex terrain of a mountain also makes it difficult to reach a favorable position for tracking. Greg Storz et al. proposed a portable FMCW harmonic radar system that is expected to be installed on a UAV [72]. The module is made more compact by reducing its power and shortening its range of operation. Although a certain detection range is sacrificed (S-band 40 m, X-band 15 m), its portability and its combination with UAVs will allow more efficient hornet tracking, which is a major development direction for the future.

### 3.2. Radio-Telemetry Tracking

Radio-telemetry technology has been widely used to study the movement behavior and spatial distribution of vertebrates [73]. In recent years, transmitters have been made light enough to be applied to insects, for example, for tracking migratory dragonflies and observing the large-scale movement of neotropical orchid bees [74,75,76]. The effective tracking range of radio telemetry is typically 100–500 m [74,77]; compared with the passive transponder of the harmonic radar, radio telemetry instead uses an active transmitter. The signal emitted by the transmitter installed on the hornet is received by the Yagi antenna, and the signal is transmitted to the receiver for numerical display. The received signal strength indicator (RSSI) value indicates the strength of the received signal. Generally, the RSSI decreases with increasing distance from the signal source. When the Yagi antenna is facing the direction of the transmitter, the RSSI is the strongest. However, the RSSI decreases gradually as the direction shifts [39]. In this way, the position and direction of the tracked hornets can be roughly determined. Researchers have tried to improve the positioning accuracy of radio telemetry by using multi-antenna arrays, successfully reducing the positioning error to less than 16 m [78].

The weight of the transmitter is a key factor affecting the tracking of hornets. Generally, passive transponders can be easily made to weigh less than 20 mg [42,79], but the lightest active transmitter currently sold is heavier than 100 mg (NanoPin, weight 130 mg, Lotek, Newmarket ON, Canada) (Table 1). As the weight of the transmitter decreases, so does the detection range. To obtain a lighter transmitter, Shearwood et al. proposed an active transmitter using bee vibration as the power source and a piezoelectric energy harvester to replace the battery, successfully reducing the weight [80]. Kumari et al. proposed a new circuit design for very high frequency (VHF) radiotelemetry [81]. This design allows the smallest tag size (5 mm × 5 mm × 2.5 mm) and tag weight (<95 mg). A unique active transmitter used for insect tracking weighs between 30 mg and 80 mg; the transmitter is able to harvest energy via a piezoelectric harvester [82].

Research shows that the weight of *V. velutina* ranges from 140 to 475 mg, and most hornets (81%) fly well when they carry a tag less than 80% of their body weight; only 14% of hornets exhibit good flight beyond this threshold [83]. Therefore, the choice of installing a transmitter on larger bodied hornets can increase the likelihood of ensuring normal flight.

Due to the portability of radio telemetry systems, a single person can carry them for tracking. However, it is difficult for the carrier to maintain the flight speed of hornets, especially on complex terrain. To this end, carrying the tracking device on a UAV and following the hornets with the UAV offers the simplest solution. Shearwood et al. used a commercial UAV equipped with a phase-controlled radar, receiver, and controller that could effectively track the bees and transmit the data to a remote base station [82]. However, due to the limitations of the transmitter, the working distance of the system is approximately 10 m. Ju et al. proposed an autonomous tracking system based on the combination of radio telemetry and a UAV [77], in which the Yagi antenna and receiver are carried on the UAV and the calculation module, flight controller, and various sensors are installed to control the position relationship between the UAV and the signal source.

On the basis of a UAV successfully carrying a radio telemetry system, Kim et al. proposed a tracking system based on multiple antennas [84]. Two omnidirectional antennas are placed in a suitable position on the ground cooperate with the UAV, and receive signals from the transmitter carried by the hornet. In this system, according to the RSSI values of the three antennas, the position of the tracked object is estimated via the triangulation method. The system can estimate the three-dimensional position information of the target to achieve more accurate tracking. The positioning error is successfully reduced from 16.4812 m to 6.8039 m. Compared with the simple use of multi-antenna arrays, the use of UAV makes the system more flexible and effectively reduces the positioning error [78].

Compared with harmonic radar, radio telemetry systems cannot intuitively display location information. However, due to the portability of its equipment, it can be carried on a UAV, and GPS can be installed on the UAV to indirectly locate the position of the hornets and obtain the location information of the nest through the position of the UAV. Of course, different regional bills have regulated the use of drones, which may affect the use and development of drones in the field of insect tracking.

## 4. Discussion

Currently, hornet-tracking technology is still not mature enough and faces many challenges. In this review, several existing hornet-tracking technologies are discussed in depth, and conclusions are drawn as follows: (1) Common hornet tags in the market influence the normal flight of hornets and cause the targets to be easily lost. Most tracking tags are suitable for simple environments in plain areas. However, the cost is low, and the operation is easy. (2) The triangulation method is a simple method for locating the approximate location of a hornet nest. However, it is necessary to consider the influence of hornets that remain on a tree or wander elsewhere. (3) Thermal imaging technology can directly determine the specific location of a hornet nest, but canopy occlusion, ambient temperature, and the operator’s angle and position can impact detection; necessarily, this technique is not applicable to a nest located in a cave. (4) Harmonic radar systems can track the movement trajectory of hornets to their nest. However, these methods are limited in complex terrain, and the system is expensive. Creating portable harmonic radar systems or improving their mobility are future development directions. (5) Radio telemetry technology is the most suitable for hornet tracking because of its portability. Moreover, these devices can be easily combined with UAVs, and adding multi-antenna arrays at the same time can effectively reduce the error. However, the weight of the transmitter is the key to successful tracking, and with the development of science and technology, there will be more lightweight transmitters in the future. (Table 2).

With the increasing harm caused by hornets, hornet-tracking technology has received increasing attention. Hornet-tracking technology will also be further improved with the development of science and technology. It is predicted that the future direction of hornet-tracking technology development will focus mainly on the following three points: (1) lightweight hornet tags and transmitters, and considering the weight of the receiver; (2) hornet tagging, triangulation, and thermal imaging technology capable of cooperating to locate nests more efficiently; and (3) combining thermal infrared, harmonic radar, and radio telemetry with UAV applications.

There are few reports on the combined use of different technologies, but the complementary advantages of different technologies may improve the efficiency and success rate of locating nests. For example, the combination of hornet tags and triangulation technology can find the area where the nest is located, and thermal imaging technology can accurately lock onto the nest; after the approximate location of the nest is determined by harmonic radar and radio telemetry technology, thermal imaging and UAV can complete the precise positioning and identification of the nest.

Choosing the appropriate hornet-tracking technology is crucial in different environments. In urban areas, where buildings and man-made structures are prevalent, radio telemetry and tag-tracking technologies are recommended. In rural environments, the open fields and fewer obstacles make triangulation and harmonic radar suitable choices. In mountainous areas, due to the complex terrain, thermal imaging technology and improved harmonic radar are more effective for locating and tracking hornet nests. By selecting the right technology based on the environmental characteristics, it is possible to more efficiently control hornet populations and protect beekeeping and ecosystems.

Finding nests by tracking hornets can effectively inhibit the continuous expansion of hornet populations, which is the most thorough and effective way to address ecological crises and dilemmas related to apiaries. Decreasing the number of invasive hornets can greatly reduce their impact on the local ecology. When the hornet nests around an apiary are removed, the health of the apiary can be restored, and the losses caused by hornet invasion can be reduced. Hornet-tracking technology will contribute significantly to the global ecology and the bee industry.

## Figures and Tables

**Table 1:** Characteristics of the lightest currently commercially available (May 2024) active radio transmitters. Data sources: accessed on 6 August 2024, Lotek (www.lotek.com); ATS (Cambridge, ON, Canada, atstrack.com); HOLOHIL (Carp, ON, Canada, www.holohil.com).

Tag Name	Weigh t (mg)	Size (mm)	Life	Pulse Rate (ppm)	Company
NanoPin	130–170	11 × 3 × 3	12–29 days	30	Lotek
T15	150	11 × 3.4 × 3.4	7–27 days	15–30	ATS
PicoPip (Ag190)	220	12 × 5 × 2	4 days	30	Lotek
LB-2X	270	11 × 5 × 2.8	8–15 days	20–120	HOLOHIL

**Table 2:** The advantages and disadvantages of the 5 tracking methods.

Name	Advantages	Disadvantages
Hornet tag	Low cost (single 0.027–0.140 USD, https://www.taobao.com, accessed on 1 August 2024), simple operation	Affects the flight of the hornets (tag weight, air resistance, rope length)
Triangulation	Low cost of equipment, simplicity to operate, can roughly locate the nest	Direction records easily contain errors (hornets handle food, flight trajectories)
Thermal imaging	Can accurately locate the nest, using the temperature difference between the nest and the environment	Affected by the environment and operation, not suitable for hidden nests
Harmonic radar	The transponder is lighter (<20 mg), the target motion trajectory can be displayed	The equipment is heavy and costly, the signal is easily blocked by the environment.
Radio-telemetry	Portable equipment, can be carried on a UAV	The transmitter is heavier for hornet, the position display is not intuitive

## Data Availability

This study did not create or analyze new data, and data sharing does not apply to this article.
